# Antimicrobial resistance in patients with decompensated liver cirrhosis and bacterial infections in a tertiary center in Northern Germany

**DOI:** 10.1186/s12876-021-01871-w

**Published:** 2021-07-20

**Authors:** Annika Hillert, Marie Schultalbers, Tammo L. Tergast, Ralf-Peter Vonberg, Jessica Rademacher, Heiner Wedemeyer, Markus Cornberg, Stefan Ziesing, Benjamin Maasoumy, Christoph Höner zu Siederdissen

**Affiliations:** 1 Department for Gastroenterology, Hepatology and Endocrinology, Hannover Medical School, Carl-Neuberg-Str. 1, 30625 Hannover, Germany; 2 Institute for Medical Microbiology and Hospital Epidemiology, Hannover Medical School, Hannover, Germany; 3 Department of Respiratory Medicine, Hannover Medical School, Hannover, Germany; 4 Antibiotic Stewardship, Hannover Medical School, Hannover, Germany; 5 Center for Individualized Infection Medicine, Hannover Medical School, Hannover, Germany; 6 Emergency Department, Hannover Medical School, Hannover, Germany; 7 Centre for Infection Research (DZIF), partner site Hannover-Braunschweig, Braunschweig, Germany

**Keywords:** Liver cirrhosis, Ascites, Antibiotic resistance, Bacterial infection, Multiresistant bacteria

## Abstract

**Background and aims:**

Bacterial infections are common in patients with decompensated liver cirrhosis and a leading cause of death. Reliable data on antibiotic resistance are required to initiate effective empiric therapy. We here aim to assess the antimicrobial resistance profile of bacteria among patients with liver cirrhosis and infection.

**Methods:**

Overall, 666 cirrhotic patients admitted to Hannover Medical School between January 2012 and April 2018 with ascites were assessed for bacterial infection. In case of infection, bacteria cultured from microbiological specimens of ascites, blood or urine were identified and analyzed for resistances against common antibiotic agents. Furthermore, analyses compared two periods of time and community-acquired vs. nosocomial infections.

**Results:**

In 281 patients with infection, microbiological sampling was performed and culture-positive results were obtained in 56.9%. Multidrug-resistant (MDR)-bacteria were found in 54 patients (19.2%). Gram-positive organisms were more common (n = 141/261, 54.0%) and detected in 116/192 culture-positive infections (60.4%). Comparing infections before and after 2015, a numerical decline for MDR-bacteria (23.8% vs. 15.6%, *p* = 0.08) was observed with a significant decline in meropenem resistance (34.9% vs. 19.5%, *p* = 0.03). MDR-bacteria were more frequent in the case of nosocomial infections. Of note, in ascites the majority of the tested bacteria were resistant against ceftriaxone (73.8%) whereas significantly less were resistant against meropenem (27.0%) and vancomycin (25.9%).

**Conclusions:**

In our tertiary center, distinct ratios of gram-positive infection with overall low ratios of MDR-bacteria were found. Adequate gram-positive coverage in the empiric therapy should be considered. Carbapenem treatment may be omitted even in nosocomial infection. In contrast, 3rd generation cephalosporins cannot be recommended even in community-acquired infection in our cirrhotic population.

**Supplementary Information:**

The online version contains supplementary material available at 10.1186/s12876-021-01871-w.

## Introduction

Bacterial infections increase the mortality of patients with liver cirrhosis up to four times [1] and are one of the leading causes of death in this population [2]. Particular hazards are numerous consecutive nosocomial infections decreasing liver transplant-free survival significantly [3]. The most frequent sites of infection are spontaneous bacterial peritonitis (SBP) and urinary tract infections (UTI) [2–4]. Patients with liver cirrhosis have a higher susceptibility for infections resulting in an increased risk for sepsis and sepsis-related mortality [5]. Early empiric antibiotic treatment can decrease the mortality of severe sepsis and is strongly recommended [6–10].

In 2018 the European Association for the Study of the Liver (EASL) published a guideline for the management of patients with decompensated cirrhosis [11]. Empirical antibiotic treatment recommendations for community-acquired SBP and bloodstream infection (BSI) are 3rd generation cephalosporins and meropenem for nosocomial SBP and BSI. Treatment recommendations for UTI include ciprofloxacin or nitrofurantoin. In addition, it is recommended to consider the local resistance profiles [11, 12]. The EASL guideline distinguishes between countries with high and low ratios of bacterial resistance [11].

International studies with individual data for single countries and regions show a prevalence of multidrug-resistant (MDR)-bacteria in patients with cirrhosis in Germany between 26% [13] and 34.1% [14] of culture-positive patients. Importantly, the prevalence varies significantly between local regions, e.g., 4.2% and 41.2% [14].

Antibiotic therapy is challenged by several trends. An increasing number of gram-positive pathogens can be found in patients with SBP [4, 15, 16] which makes 3rd generation cephalosporins less effective [17]. Additionally, due to frequent hospitalization and regular use of antibiotics in patients with liver cirrhosis, cirrhotic patients have a higher risk for developing infections caused by MDR-bacteria [18]. Those infections are associated with a higher incidence of treatment failure, septic shock, and hospital mortality ][13, 14, 18] and are increasing dramatically according to several studies [4, 18, 19].

The EASL recommendations for the antibiotic treatment of patients with liver cirrhosis are based on pan-European studies on pathogens and their antibiotic resistance and aim to provide valid recommendations across Europe. However, given the changing epidemiology of pathogens and resistances as well as local regional differences, it may be better to rely on local data than global recommendations.

With the present study, we aim to present a big database of frequently found pathogens and their resistances in microbiology samples taken during SBP, BSI, and UTI from patients with decompensated cirrhosis and question whether the EASL recommendations are adequate in our region. In addition, further analyses distinguish between two periods of time to assess trends in MDR-infection and between community-acquired vs. nosocomial infections. Based on these findings we aim to provide empiric treatment recommendations for our tertiary center in Northern Germany.

## Material and methods

### Patient cohort

**Fig. 1:**
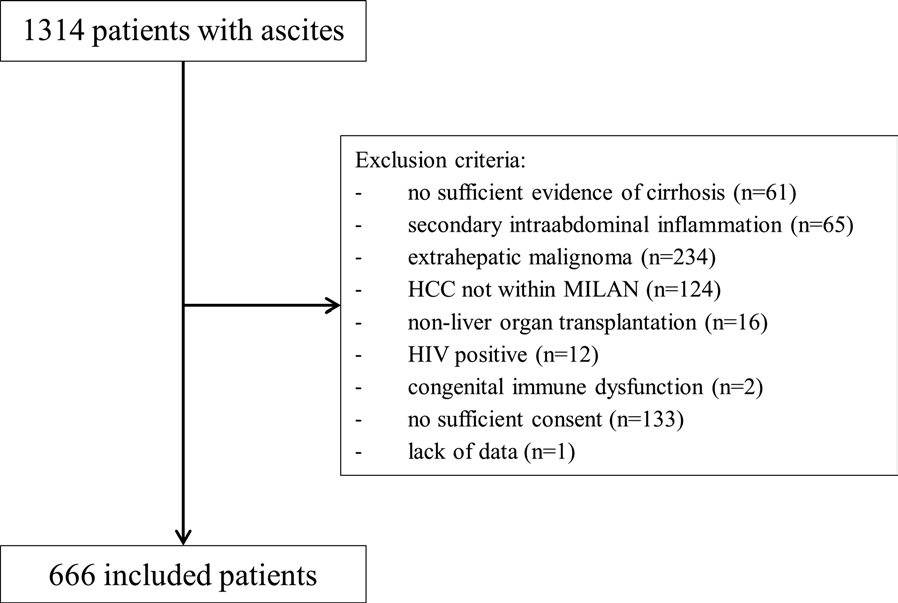
Selection of patients. This figure depicts the selection of patients. *HCC* hepatocellular carcinoma, *HIV* human immunodeficiency virus

The patients were recruited from the Hannover Ascites Cohort, which considered all consecutive patients with liver cirrhosis who underwent paracentesis during their hospital stay at the Hannover Medical School between January 2012 and April 2018. The patients were automatically identified retrospectively by the Enterprise Clinical Research Data Warehouse (ECRDW) to minimize a potential selection bias. Overall, 1314 patients were screened and 666 patients were included after manual assessment of the patient’s charts (Fig. 1). Routine laboratory data were collected using the ECRDW. Routine clinical data including the date of infection and antibiotic susceptibility testing (AST) results were collected manually from the patients’ files. Diagnosis of liver cirrhosis was based on ultrasound, FibroScan (≥ 14.5 kPa), biochemical results, and/or liver biopsy.

Exclusion criteria were lack of evidence of cirrhosis, extrahepatic malignancy, hepatocellular carcinoma beyond the Milan criteria, secondary intraabdominal inflammation, infection with HIV, congenital immune dysfunction, non-liver organ transplantation, lack of data, and absence of written informed consent.

### Ethics

The local ethics committee of Hannover Medical School approved the study (ethic no. 7935_BO_K_2018 from June 22nd 2018) and patients provided written informed consent for the scientific use of their clinical data. The study was conducted according to the Declaration of Helsinki.

### Data assessment

The observation period included the complete hospital stay until discharge, transfer to another hospital or nursing home, liver transplantation, or death. Infections were diagnosed by the attending physician or in case of uncertainty by infectiologists (BM, MC, JR, CHZS) based on clinical symptoms and the following laboratory results:

- SBP: ≥ 500 nuclei containing cells/µl ascites fluid [20]
- BSI: positive blood cultures
- UTI: leukocyturia and/or positive urine cultures with significant germination number (≥ 10^5^ CFU/ml urine).

In this study, ascites and blood were considered sterile. Therefore, all the pathogens found in ascites or blood during infection were included in the analyses. Pathogens found in urine during infection were only included if at least 10^5^ CFU/ml urine were detected except for *Escherichia coli* according to local guidelines [21].

Infection was classified based on the time from admission as community-acquired (< 48 h) or nosocomial (> 48 h) [22]. If the patient was hospitalized within the previous three months, infections in the first 48 h after admission were classified as nosocomial.

Pathogens were counted once per site and patient only. In case, a pathogen was redetected, only the first Antimicrobial Susceptibility Testing was included in our study. For easier reading, a sample with a detected pathogen is called positive.

The AST was evaluated according to European Committee on Antimicrobial Susceptibility Testing (EUCAST) international guidelines and expert microbiologists (RV, SZ). Resistant results include intrinsic resistances as well as acquired resistances. Intermediate results were stated as resistant.

Definitions for multiresistant bacteria are not consistent. For better distinction the existence of one of the following resistances classified the pathogen as severe resistant:

- Vancomycin- or Linezolid-resistant *Enterococcus*
- Oxacillin-/Methicillin-resistant *Staphylococcus aureus* (ORSA/MRSA)
- Multiresistant gram-negative germ (3MRGN/4MRGN) according to the recommendation of the German commission of hospital hygiene and infection prevention (KRINKO) [23] (Additional file 1: Table 1).

*Enterobacteriaceae*, *Staphylococcus aureus*, *Enterococcus spp.,* and *Acinetobacter spp.* were classified as multidrug-resistant (MDR) if the isolate is non-susceptible to at least 1 agent in ≥ 3 antimicrobial categories [24]. In our study, all pathogens with a severe resistance met MDR-criteria as well. Additional file 2: Table 2 shows the antimicrobial categories and agents used for the definition of MDR.

### Study design

Three main analyses are part of this study. In the first analysis, the development of antibiotic resistance over time was observed. For this part, the cohort was divided into two groups. The first one (“period 1”) contains the samples that were taken during an infection between January 1st 2012 and December 31st 2014. The second period (“period 2”) includes the samples that were taken between January 1st 2015 and April 30th 2018. The analysis compares the number of severe resistances, MDR, and the ratios of resistances against common antibiotics.

The second analysis presents the ratios of bacteria with resistance against common antibiotics in SBP, BSI, and UTI. In a further analysis, these samples are subdivided into community-acquired and nosocomial infections.

The third analysis evaluates the treatment options for SBP, BSI and UTI recommended by EASL. For this part, we analyzed the ratios of patients with resistance against the recommended antibiotics in mono- and combination-therapy. Suboptimal antibiotic selection was assumed in patients if at least one pathogen was resistant to all applied antibiotic agents.

### Statistics

Statistical analyses were performed with SPSS (IBM SPSS Statistics 26). Continuous data were analyzed with unpaired t-test and categorical data with Fisher´s exact test.

## Results

### Baseline characteristics

Overall, 666 patients were included in this study. The majority (64.3%) was male and the median age was 56.7 (17–87) years. The major causes of liver cirrhosis were alcohol (50.5%) and hepatitis C virus infection (13.8%) with a median MELD-Score of 17.6. In half of the patients, at least one infection was diagnosed. The most common infection was SBP followed by UTI and BSI (Table 1).

**Table 1:** Patients’ characteristics

Variable	All patients	Sampled patients*^1^	Period 1 (01/2012–12/2014)*^1^	Period 2 (01/2015–04/2018)*^1^	*p* value*^2^
Total number of patients	666	280	126	155	
*Sex*					
Female (n, %)	238 (35.7%)	97 (34.6%)	44 (34.9%)	53 (34.4%)	0.93
Male (n, %)	428 (64.3%)	183 (65.4%)	82 (65.1%)	101 (65.6%)	0.93
Age (years)	56.7 (17–87)	57.3 (17.8–85.8)	56.9 (21.5–81)	57.7 (17.8–85.8)	0.54
Days in hospital	15.5 (1–126)	23.0 (1–126)	24.5 (2–97)	22.0 (1–126)	0.11
90-day mortality*^3^	148 (22.2%)	89 (31.8%)	38 (30.2%)	51 (33.1%)	0.60
Etiology of liver cirrhosis*^4^					
Alcohol-related (n, %)	336 (50.5%)	130 (46.4%)	56 (44.4%)	74 (48.1%)	0.54
HCV (n, %)	92 (13.8%)	37 (13.2%)	20 (15.9%)	17 (11.0%)	0.24
HBV (n, %)	29 (4.4%)	10 (3.6%)	4 (3.2%)	6 (3.9%)	0.75
NASH (n, %)	45 (6.8%)	27 (9.6%)	11 (8.7%)	16 (10.4%)	0.77
Other (n, %)	226 (33.9%)	102 (36.4%)	45 (35.7%)	57 (37.0%)	0.82
*Laboratory values at admission*					
MELD	17.6 (6.43–40)	19.8 (7.9–40.0)	19.2 (8.6–40.0)	19.9 (7.9–40.0)	0.41
INR	1.42 (0.97–5.64)	1.5 (1.0–5.6)	1.5 (1.1–3.4)	1.5 (1.0–5.6)	0.09
Creatinine (µmol/l)	109.5 (30–878)	124.0 (32.0–878.0)	116.0 (40.0–691.0)	126.0 (32.0–878.0)	0.52
Thrombocytes (× 10^3^/µl)	117 (15–813)	113.5 (15.0–813.0)	110.5 (15.0–490.0)	123.0 (17.0–813.0)	0.28
CRP (mg/l)	24 (0.4–433)	28.1 (0.9–433.0)	26.0 (2.0–433.0)	30.8 (0.9–266.0)	0.78
Bilirubin (µmol/l)	42 (3–786)	54.0 (3.0–786.0)	54.0 (4.0–786.0)	54.0 (3.0–676.0)	0.85
Sodium (mmol/l)	134 (115–149)	134.0 (115.0–149.0)	134.0 (117.0–145.0)	134.0 (115.0–149.0)	0.95
Leucocytes (× 10^3^/µl)	7.6 (1.1–83.4)	8.5 (1.9–83.4)	8.6 (2.3–83.4)	8.5 (1.9–35.5)	0.90
Patients with SBP (n, %)	262 (39.3%)	205 (73.2%)	91 (72.5%)	114 (77.3%)	0.74
Patients with BSI (n, %)	49 (7.4%)	49 (17.5%)	28 (22.2%)	21 (13.6%)	0.06
Patients with UTI (n, %)	153 (23.0%)	93 (33.2%)	37 (29.4%)	56 (36.4%)	0.22
Patients with at least one infection (n, %)	354 (53.2%)				
Patients with one of the three infection (n, %)	256 (38.4%)	219 (78.2%)	100 (79.4%)	119 (77.3%)	0.67
Patients with two of the three infections (n, %)	86 (12.9%)	55 (19.6%)	22 (17.5%)	33 (21.4%)	0.41
Patients with all three infections (n, %)	12 (1.8%)	6 (2.1%)	4 (3.2%)	2 (1.3%)	0.28
Patients with microbiological sampling during infection (n, %)	281 (42.2%)				
Patients with at least one positive microbiological sample (n, %)	160 (24.0%)	159 (56.8%)	76 (60.3%)	83 (53.9%)	0.28

*^1^Only patients with microbiological sampling during infection included. One patient was excluded because the time of his infection overlapped the cutoff date for both periods

*^2^Comparison of the two groups “period 1” and “period 2”

*^3^The 90 days were counted starting from the end of hospitalization

*^4^70 patients (32 sampled patients, 14 in period 1, 18 in period 2) had more than one etiology of cirrhosis

Categorical variables are expressed as the number and percentage of the total study group or subgroup. Continuous variables are expressed as median and range

*HCV* hepatitis C virus, *HBV* hepatitis B virus, *NASH* nonalcoholic steatohepatitis, *MELD* model for end-stage liver disease, *INR* International normalized ratio of prothrombin time, *CRP* C-reactive protein, *SBP* spontaneous bacterial peritonitis, *BSI* bloodstream infection, *UTI* urinary tract infection; n, number of patients

Microbiological sampling was performed in 281 patients, 56.9% of them had at least one culture-positive sample. Samples, in which more than one pathogen was detected, were found in 46 patients (16.4%). Pathogens with severe resistance as defined above were found in twelve patients (4.3%). MDR-bacteria were found in 54 patients (19.2%).

### Frequency of pathogens

Overall, 261 pathogens were found in 192 culture-positive infections of 160 patients. If pathogens were detected at more than one site of a patient, the detected species were the same in 60.0% of the patients with SBP and BSI (n = 6/10), 15.4% of the patients with SBP and UTI (n = 2/13), 28.6% of patients with BSI and UTI (n = 4/14) and 20.0% of patients with evidence of infection at all three infection sites (n = 1/5). Fifty-four infections were polymicrobial, most commonly containing *E. faecalis* (31.5%, n = 17), *E. faecium* (24.1%, n = 13), *E. coli* (22.2%, n = 12) and *S. aureus* (14.8%, n = 8) (Additional file 3: Table 3).

The most frequent pathogen was *Escherichia coli*. The second and third most frequent pathogens were the gram-positive pathogens *Enterococci* and *Staphylococci*. Of note, more than half of the detected pathogens were gram-positive organisms (n = 141 of 261, 54.0%), found in 116 of 192 culture-positive infections (60.4%). Of all *Enterobacteriaceae* found in the samples, 49.1% (n = 28/57) were classified as MDR, and 16.7% (n = 1/6) of *Acinetobacter spp.* Of the 28 *Staphylococcus aureus* 35.7% (n = 10) were classified as MDR (MRSA) and 38.9% (n = 21/54) of *Enterococcus spp.* In total, 23.0% (n = 60/261) of all detected pathogens were MDR, 23.3% of them (n = 14/60) had a severe resistance (Table 2).

**Table 2:** Most frequent pathogens.

Pathogen	No. (%) of pathogens			
	Total	Ascites	Blood	Urine
	(n = 261)	(n = 90)	(n = 63)	(n = 108)
*Gram-negative organisms*				
All	67 (25.7)	21 (23.3)	17 (27.0)	29 (26.9)
*Escherichia coli*	35 (13.4)	13 (14.4)	6 (9.5)	16 (14.8)
*Klebsiella pneumoniae*	14 (5.4)	4 (4.4)	5 (7.9)	5 (4.6)
Other	18 (6.9)	4 (4.4)	6 (9.5)	8 (7.4)
*Gram-positive organisms*				
All	141 (54.0)	65 (72.2)	42 (66.7)	34 (31.5)
*Staphylococcus aureus*	28 (10.7)	14 (15.6)	11 (17.5)	3 (2.8)
*Enterococcus faecalis*	27 (10.3)	6 (6.7)	4 (6.3)	17 (15.7)
*Enterococcus faecium*	23 (8.8)	9 (10.0)	6 (9.5)	8 (7.4)
*Staphylococcus epidermidis*	23 (8.8)	7 (7.8)	14 (22.2)	2 (1.9)
*Staphylococcus haemolyticus*	10 (3.8)	8 (8.9)	2 (3.2)	0 (0.0)
Other	30 (11.5)	21 (23.3)	5 (7.9)	4 (3.7)
*Fungi*				
All	42 (16.1)	4 (4.4)	4 (6.3)	34 (31.5)
*Candida albicans*	21 (8.0)	2 (2.2)	2 (3.2)	17 (15.7)
Other	21 (8.0)	2 (2.2)	2 (3.2)	17 (15.7)
Other	11 (4.2)	0 (0.0)	0 (0.0)	11 (10.2)
*Multidrug-resistant bacteria (MDR-bacteria)*				
All	60 (23.0)	22 (24.4)	14 (22.2)	24 (22.2)
Multiresistant gram-negative germ (3MRGN)	6 (2.3)	1 (1.1)	1 (1.6)	4 (3.7)
Oxacillin-/methicillin-resistant *Staphylococcus aureus* (ORSA/MRSA)	5 (1.9)	2 (2.2)	2 (3.2)	1 (0.9)
Vancomycin-resistant *Enterococcus*	2 (0.8)	0 (0.0)	0 (0.0)	2 (1.9)
Linezolid-resistant *Enterococcus*	1 (0.4)	0 (0.0)	0 (0.0)	1 (0.9)

### Changes in antibiotic resistance over time

To assess the development of antibiotic resistance over time, all 280 patients were compared depending on the date of infection. One hundred and twenty-six patients were allotted for period 1 (2012–2015) and 154 patients for period 2 (2015–2018). The baseline parameters of the two study groups are depicted in Table 1.

**Fig. 2:**
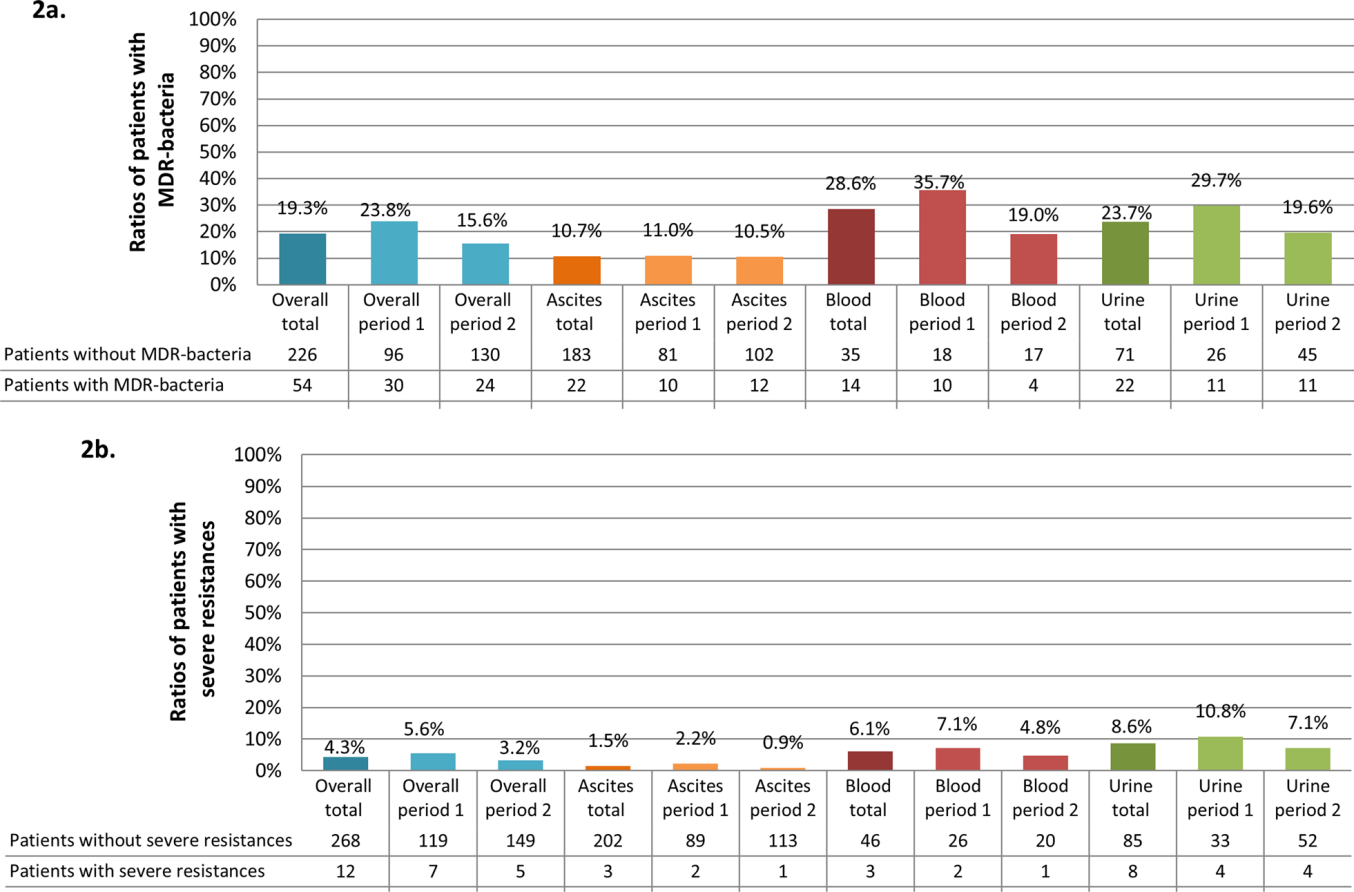
Ratios of patients with MDR-bacteria (**a**) and bacteria with severe resistances (**b**). **a** Presents the ratios of patients with MDR-bacteria. **b** Shows the ratio of patients with severe resistances. The group “overall” does not comply with the sum of the subgroups, because some patients had more than one infection. MDR: multidrug-resistance

In both periods more than half of the patients had at least one positive sample (60.3% in period 1 vs. 53.9% in period 2, *p* = 0.28). In period 2 less patients had MDR-bacteria (23.8% vs. 15.6%, *p* = 0.08) (Fig. 2a). The ratio of patients with severe resistances was slightly higher in period 1 compared to period 2 (5.6% vs. 3.2%, *p* = 0.34) (Fig. 2b). Across the tested antibiotics, no major shift in resistances could be confirmed, except for meropenem resistance, which significantly decreased (34.9% vs. 19.5%, *p* = 0.03) (Table 3).

**Table 3:** Susceptibility of pathogens in period 1 and period 2

Antibiotic agent	Period 1 (01/2012–12/2014)				Period 2 (01/2015–04/2018)				*p* value
	Susceptible		Resistant		Susceptible		Resistant		
Overall	n	%	n	%	n	%	n	%	
Ampicillin	31	34.4	59	65.6	33	32.4	69	67.6	0.76
Ceftriaxone	32	34.0	62	66.0	26	26.0	74	74.0	0.22
Ciprofloxacin	33	62.3	20	37.7	48	68.6	22	31.4	0.47
Meropenem	56	65.1	30	34.9	62	80.5	15	19.5	0.03
*Ascites*									
Ampicillin	15	41.7	21	58.3	14	29.8	33	70.2	0.26
Ceftriaxone	13	36.1	23	63.9	8	18.2	36	81.8	0.07
Ciprofloxacin	13	68.4	6	31.6	23	63.9	13	36.1	0.74
Meropenem	23	67.6	11	32.4	31	77.5	9	22.5	0.34
*Blood*									
Ampicillin	9	27.3	24	72.7	5	22.7	17	77.3	0.71
Ceftriaxone	10	27.8	26	72.2	6	27.2	16	72.7	0.97
Ciprofloxacin	11	61.1	7	38.9	10	58.8	7	41.2	0.89
Meropenem	19	57.6	14	42.4	15	75.0	5	25.0	0.20
*Urine*									
Ampicillin	7	33.3	14	66.7	14	42.4	19	57.6	0.50
Ceftriaxone	9	40.9	13	59.1	12	35.3	22	64.7	0.67
Ciprofloxacin	9	56.3	7	43.8	15	88.2	2	11.8	0.06
Meropenem	14	73.7	5	26.3	16	94.1	1	5.9	0.18

N: number of susceptible or resistant results

### Antibiotic resistance in common locations for infection

Pathogens were detected in more than half of the urine samples taken during UTI (66.9%, n = 83/124) and blood cultures taken during BSI (56.1%, n = 55/98) but in less than a quarter of ascites samples taken during SBP (23.2%, n = 72/310). The highest ratio of MDR-bacteria was detected in ascites samples (ascites: 24.4%, n = 22/90; urine: 22.2%, n = 24/108; blood: 22.2%, n = 14/63). Pathogens with severe resistance were more likely found in urine samples (7.4%, n = 8/108; blood: 4.8%, n = 3/63; ascites: 3.3%, n = 3/90) (Additional file 4: Table 4).

Table 4 shows the AST results for commonly used antibiotics for the three different types of infection. In ascites the majority of the tested bacteria were resistant against ceftriaxone (73.8%), whereas only around a quarter was resistant against meropenem (27.0%) and vancomycin (25.9%). Piperacillin/tazobactam showed similar effectiveness as meropenem with only 34.5% (n = 29/55) of the tested bacteria being resistant. Bacteria found in blood were slightly more resistant to commonly used antibiotics. In urine, 23 of the detected pathogens had resistances against at least four antibiotics commonly used for UTI (susceptibility and resistances for distinct antibiotics and combinations are depicted in Table 4).

**Table 4:** Susceptibility of pathogens in different infections for antibiotics commonly used in mono- or combination therapy

Antibiotic agent	SBP				BSI				UTI			
	S		R		S		R		S		R	
	n	%	n	%	n	%	n	%	n	%	n	%
Ampicillin/sulbactam	51	60.7	33	39.3	31	56.4	24	43.6	–	–	–	–
Piperacillin/tazobactam	55	65.5	29	34.5	30	53.6	26	46.4	36	65.5	19	34.5
Amoxicillin-/clavulanic acid	–	–	–	–	–	–	–	–	12	46.2	14	53.8
Cefpodoxime	–	–	–	–	–	–	–	–	18	40.0	27	60.0
Cefuroxime	37	46.3	43	53.8	23	45.1	28	54.9	–	–	–	–
Ceftriaxone	21	26.3	59	73.8	16	27.6	42	72.4	22	38.6	35	61.4
Fosfomycin	–	–	–	–	–	–	–	–	15	88.2	2	11.8
Levofloxacin	–	–	–	–	–	–	–	–	32	59.3	22	40.7
Ciprofloxacin	–	–	–	–	–	–	–	–	25	73.5	9	26.5
Meropenem	54	73.0	20	27.0	34	64.2	19	35.8	31	83.8	6	16.2
Vancomycin	63	74.1	22	25.9	40	70.2	17	29.8	–	–	–	–
Cotrimoxazole	–	–	–	–	–	–	–	–	27	48.2	29	51.8
Nitrofurantoin	–	–	–	–	–	–	–	–	10	100	0	0.0
Ampicillin/sulbactam/vancomycin	76	90.5	8	9.5	49	89.1	6	10.9	27	96.4	1	3.6
Piperacillin/tazobactam/vancomycin	79	94.0	5	6.0	48	85.7	8	14.3	26	100	0	0.0
Meropenem/vancomycin	73	100	0	0.0	52	98.1	1	1.9	8	100	0	0.0
Ceftriaxone/vancomycin	72	91.1	7	8.9	52	92.9	4	7.1	26	96.3	1	3.7

*SBP* spontaneous bacterial peritonitis, *BSI* bloodstream infection, *UTI* urinary tract infection, *n* number of susceptible or resistant results, *S* susceptible, *R* resistant

For further analyses, the SBP and UTI cohorts were divided into community-acquired and nosocomial infections. This separation was not possible for BSI, because only three patients had a community-acquired infection. In ascites the ratio of patients with positive cultures was significantly higher in nosocomial SBP versus community-acquired infection (36.9% vs. 20.0%, *p* = 0.04). MDR-bacteria were slightly more frequent in nosocomial episodes (11.5% vs. 7.5%, *p* = 0.46). In addition, bacteria with severe resistances were only found in patients with nosocomial episodes of SBP. Similarly, for UTI the patients with nosocomial infections were more likely to have positive cultures compared with community-acquired UTI (79.5% vs. 72.7%, *p* = 0.61). Moreover, MDR-bacteria were also more frequent in patients with nosocomial UTI (24.1% vs. 18.2%, *p* = 0.66). However, patients with severe resistant pathogens were found equally in both groups (nosocomial vs. community-acquired, 9.1% vs. 8.4%, *p* = 0.94). No major differences in resistances between community-acquired and nosocomial infections were found (Additional file 5: Table 5).

### Application of treatment guidelines

To assess the effectivity of the EASL guidelines for the treatment of UTI, BSI or SBP at our center, we compared our local resistances with the EASL recommendations. For community-acquired SBP or septic UTI the recommended antibiotic regimen are 3rd generation cephalosporines or piperacillin/tazobactam [11]. In our cohort 61.3% of all culture-positive patients (n = 98 of 160) had at least one pathogen resistant to ceftriaxone. This ratio was higher in patients with BSI (71.4%, n = 35 of 49) and SBP (66.7%, n = 46 of 69) compared to patients with UTI (43.2%, n = 32 of 74). The ratio of culture-positive patients with at least one pathogen resistant to piperacillin/tazobactam was lower with 38.8% (n = 62/160, BSI: 51.0%, n = 25/49; SBP: 36.2%, n = 25/69; UTI: 24.3%, n = 18/74). Meropenem resistant pathogens were found in 23.1% culture-positive patients (n = 37/160, BSI; 36.7%, n = 18/49, SBP: 24.6%, n = 17/69, UTI: 8.1%, n = 6/74). In UTI the following ratios of patients with resistant pathogens were detected: ciprofloxacin 12.2% (n = 22/74), cotrimoxazol 37.8% (n = 28/74), nitrofurantoin 0.0% (n = 0/74) and fosfomycin 2.7% (n = 2/74).

For nosocomial infection, combination therapy is recommended if risk factors are present, usually a carbapenem plus vancomycin, daptomycin, or linezolid. In our cohort, the combination therapy with meropenem plus vancomycin showed susceptibility in all patients with culture-positive SBP and UTI, except for one patient with BSI (2.0%, n = 1/49). Combination therapy with ampicillin/sulbactam plus vancomycin showed corresponding resistances in 11.6% of patients with culture-positive SBP (n = 8/69) and 12.2% in patients with BSI (n = 6/49). Therapy with piperacillin/tazobactam plus vancomycin had corresponding resistances in 16.3% of patients with BSI (n = 8/46), 7.2% of patients with culture-positive SBP (n = 5/69) and 0.0% of patients with culture-positive UTI (n = 0/74). The combination of ceftriaxone plus vancomycin was affected by corresponding resistances in 7.5% (n = 12/160) of patients with culture-positive infections. The highest ratio was shown in patients with culture-positive SBP (10.1%, n = 7/69) followed by BSI (8.2%, n = 4/49) and UTI (1.4%, n = 1/74).

## Discussion

Cirrhosis is associated with a high frequency of infections, most notably SBP and UTI. To decrease resulting complications and mortality, early diagnosis and adequate empiric treatment based on the knowledge of local antibiotic resistances are necessary.

We here show that (1) a distinct ratio of infections was caused by gram-positive bacteria, (2) 3rd generation cephalosporins, commonly recommended as first-line therapy, were subject to a high ratio of antimicrobial resistance in our center, even in community-acquired infections, (3) the overall frequency of infections caused by MDR-bacteria was declining and resistance to meropenem was declining in particular and (4) other antibiotics seemed to be almost equally effective as meropenem even for the treatment of nosocomial infection, in particular, if combination therapy with vancomycin was applied.

In our study, the 90-day mortality was 30.5% for patients with infection (n = 108/354). This ratio is a little bit lower compared to other studies, whereby there is a big range (1). The 90-day mortality differed according to the time of infection. Whereas patients with nosocomial infection showed a 90-day mortality of 34.4% (n = 99/288), the ratio was significantly lower for patients with community-acquired infection (13.2%, n = 7/53, *p* = 0.002).

Whereas in the past the majority of infections in cirrhotic patients were caused by gram-negative bacteria [25, 26], we currently observe a shift to the gram-positive bacteria. Several reasons have been linked to this shift: the more frequent use of invasive procedures [4], the frequent use of proton pump inhibitors (PPI) with a consecutive shift in intestinal microbiota [27], and alcohol intake with subsequent failure of the intestinal barrier and bacterial translocation [28]. In our cohort 542 patients (81.4%) had PPI in their daily medication and 336 patients (50.5%) had alcohol-related liver damage. However, no difference in ratios of gram-positive bacteria were found (ASH vs. non-ASH: 53.5%, n = 69/129 vs. 54.5%, n = 72/132; *p* = 0.86. PPI vs. non-PPI: 54.3%, n = 127/234 vs. 50.0%, n = 12/24; *p* = 0.69). Gram-positive bacteria were especially frequent (72.2%) in ascites which is in line with recent studies [15, 17, 29]. This is important to acknowledge as single antibiotic therapy will usually fail in our cohort, and the addition of vancomycin drastically increases the chances of effective antibiotic therapy. However, when interpreting these data, it has to be considered, that in some cases the detected pathogens belonged to the group of facultative pathogenic bacteria. Thus, we have to acknowledge, that the findings of e.g., certain gram-positive pathogens may be due to contamination in some cases while the true origin of infection is to be found elsewhere.

Third-generation cephalosporins are a first-line recommendation for community-acquired SBP [30, 31]. Our results showed high ratios of resistances against ceftriaxone in patients with SBP (73.8%). Resistance was more frequent in nosocomial episodes (75.0%) compared to community-acquired episodes (62.5%) and increased over time by 28.0% (from 63.9% in period 1–81.8% in period 2, *p* = 0.07). This finding is in line with other recent reports [32–35]. Thus, special consideration should be given to the use of 3^rd^ generation cephalosporins according to local resistance profiles.

Resistance is usually higher in nosocomial infections than in community-acquired infections [18, 33]. We did not find a significant difference in resistances between these two groups. However, similar to the results of Umgelter et al. [32], the majority of patients (81.4%, n = 288/354) in our study were classified as having a nosocomial infection. Other studies report lower rates [36, 37]. The high rate of nosocomial infection is well explained by the fact, that we provide tertiary care for patients with liver diseases. Thus, patients are more likely to be referred from other hospitals and tend to have more advanced liver disease (median MELD 17.6). The majority of classifications as nosocomial infection were caused by recent hospitalizations for various reasons—a common event in patients with decompensated cirrhosis [38]. Only 20 patients were directly admitted for infection, whereas the vast majority came to our hospital for ascitic decompensation or evaluation of transjugular intrahepatic portosystemic shunt placement or liver transplantation and infection was diagnosed on further work-up. It should be noted, that repeated hospitalization is a common event in cirrhotic patients. Therefore, per definition community-acquired infection may be caused by a different group of bacteria, which may influence resistance weights.

Based on our results first-line empiric therapy with piperacillin/tazobactam plus vancomycin for nosocomial SBP and UTI and meropenem plus vancomycin for nosocomial BSI might be the best choice for our center. However, this assumption can’t simply be transferred to other hospitals due to regional differences in resistance prevalence.

Due to repeated hospitalizations and exposure to antibiotics, cirrhotic patients are increasingly challenged by MDR-infections [39]. Comparison between different studies is difficult due to inconsistent definitions of multiresistance. We used the definition of Magiorakos et al. [24] as well as specific resistances like oxacillin-resistance in *Staphylococcus aureus* to describe our cohort. Overall, 60 (31.3%) of 192 culture-positive infections were caused by MDR-bacteria. This ratio was similar in all culture sites (SBP: 31.8%, n = 22/69; BSI: 28.6%, n = 14/49; UTI: 32.4%, n = 24/74) and is similar to results from other recent studies [18, 40]. Compared to other German centers, we found no VRE in ascites during SBP [35, 41], despite high ratios of *Enterococcus spp.*, and could not detect an increase of MDR-bacteria [42]. However, parts of the gram-positive pathogens must be considered as possible contamination. Analyzing only patients with possible MDR-bacteria (*Enterobacteriaceae*, *Staphylococcus aureus*, *Enterococcus spp.* or *Acinetobacter spp.*) the ratio of MDR-classified pathogens decreased over time (data not shown). In patients with gram-negative pathogens ratio of MDR-bacteria did not change over time (data not shown). Therefore, the missing increase of MDR-bacteria in this study can’t be fully explained by contamination. For single antibiotics, the resistance against ceftriaxone was distinctly higher in our cohort [17, 29], whereas vancomycin resistance distinctly was lower [17]. In addition to local differences, some of the different findings may be explained by the time point of sampling, as antibiotic resistance may shift over time. Overall, the present data underline the recommendation to assess and track local rather than national resistance profiles.

In addition to the analyzed infections, pneumonia is common in patients with liver cirrhosis as well [2]. However, in this study cohort, respiratory material was infrequently collected or consisted mainly of sputum and the culture results were commonly positive for several bacteria of the oral flora. Unfortunately, these conditions were impeding analysis for patients with pneumonia.

This study has some limitations. First, it is based on retrospective data. As retrospective estimation of pathogenicity is difficult, we did not exclude pathogens that may be cultured due to contamination (i.e., *coagulase-negative staphylococci*, *Corynebacterium*, *Propionibacterium*, or *Bacillus spp.*). As mentioned before, this can partly explain the high ratio of gram-positive bacteria compared to other studies [33]. In addition, ratios of MDR-bacteria and other resistances could be stated too low due to including non-pathogenic germs. Second, there is a lack of microbiology sampling in many patients. Third, most patients had nosocomial infections by our definition, which makes the comparison between community-acquired and nosocomial infections difficult. Nevertheless, due to the large number of included patients and observed episodes of infections the results can help to adapt guidelines for empirical antibiotic treatment.

In conclusion, our study shows that (1) a distinct number of infections was caused by gram-positive bacteria, (2) 3rd generation cephalosporins had high ratios of antibiotic resistance at our center, (3) no increase in infections with MDR-bacteria occurred between 2012 and 2018, (4) for our center the first-line empiric therapy for patients with nosocomial SBP was sufficient with piperacillin/tazobactam, even despite declining resistances to meropenem. Thus, we can confirm the higher frequency of gram-positive pathogens with the consequence of gram-positive antibiotic coverage. However, our data shows a disparity for the first-line recommendation of third-generation cephalosporins in community-acquired infection and meropenem in nosocomial infections by the EASL guideline. Nevertheless, data regarding antibiotic resistance seems to show huge differences in different countries as well as in different clinics and regions of one country. Therefore, the results of this local study can’t easily be transferred to other clinics or countries and local surveillance is strongly recommended.

## Supplementary Information

**Additional file 1.Supplemental table 1:** Classification of multiresistant gram-negative bacilliform bacteria based on their phenotypic resistance characteristics.

**Additional file 2. Supplemental table 2:** Antimicrobial categories and agents used to define MDR.

**Additional file 3. Supplemental table 3:** Pathogens in polymicrobial infections.

**Additional file 4. Supplemental table 4:** Overview of the frequency and locations of infections.

**Additional file 5. Supplemental table 5:** Susceptibility of pathogens in community-acquired vs. nosocomial infections.

## Data Availability

A data set sufficient to reproduce all main and supplementary figures is part of the manuscript or has been uploaded as supporting information. Due to ethical reasons, there are restrictions on sharing a complete data set of individual patients of this study publicly. The authors acknowledge the risk of private information being revealed by the public data disclosure, as the data underlying this study contain potentially identifiable and sensitive patient information. Therefore, the authors agreed that restrictions on sharing this study data should apply and data should be handled with care in order to protect the patients’ confidentiality. Requests for access to a full de-identified data set of the study by qualified investigators can be addressed to the local ethics committee of Hannover Medical School (Ethikkommission@mh-hannover.de) or to the corresponding author.

## Abbreviations

AST: Antibiotic susceptibility testing
BSI: Blood stream infection
CFU: Colony forming unit
EASL: European Association for the Study of the Liver
ECRDW: Enterprise clinical research data warehouse
EUCAST: European Committee on Antimicrobial Susceptibility Testing
HBV: Hepatitis B virus
HCC: Hepatocellular carcinoma
HCV: Hepatitis C virus
HIV: Human immunodeficiency virus
KRINKO: Commission of hospital hygiene and infection prevention
MDR: Multidrug-resistance
NASH: Nonalcoholic steatohepatitis
ORSA/MRSA: Oxacillin-/Methicillin-resistant *Staphylococcus aureus*
SBP: Spontaneous bacterial peritonitis
UTI: Urinary tract infection
3MRGN: Multiresistant gram-negative germ
