# Supplementary material for: Antimicrobial resistance in patients with decompensated liver cirrhosis and bacterial infections in a tertiary center in Northern Germany
Source: BMC Gastroenterol. 2021 Jul 20;21:296. doi: 10.1186/s12876-021-01871-w (PMC8290615; doi:10.1186/s12876-021-01871-w)
Supplement: Supplementary file 1 — Additional file 1.Supplemental table 1: Classification of multiresistant gram-negative bacilliform bacteria based on their phenotypic resistance characteristics. [file 12876_2021_1871_MOESM1_ESM.docx]

**Supplemental table 1:** Classification of multiresistant gram-negative bacilliform bacteria based on their phenotypic resistance characteristics

| Antimicrobial category | Lead antimicrobial | Enterobacterales | | Pseudomonas aeruginosa | | Acinetobacter baumannii | |
| --- | --- | --- | --- | --- | --- | --- | --- |
|  |  | 3MRGN^1^ | 4MRGN^2^ | 3MRGN^1^ | 4MRGN^2^ | 3MRGN^1^ | 4MRGN^2^ |
| Acylureidopenicillin | Piperacillin | R | R | Just one of the four antimicrobial categories effective (susceptible) | R | R | R |
| 3rd. / 4th. generation cephalosporin | Cefotaxime and/or Ceftazidime | R | R |  | R | R | R |
| Carbapenem | Imipenem and/or Meropenem | S | R |  | R | S | R |
| Fluoroquinolone | Ciprofloxacin | R | R |  | R | R | R |
| ^1^ 3MRGN (**m**ulti**r**esistant **g**ram-**n**egative bacilli with resistance against **3** of 4 of the antimicrobial categories)  ^2^ 4MRGN (**m**ulti**r**esistant **g**ram-**n**egative bacilli with resistance against **4** of 4 of the antimicrobial categories) | | | | | | | |

R: resistant or intermedium susceptible, S: susceptible, Quoted from (27)
