# Supplementary material for: Antimicrobial resistance in patients with decompensated liver cirrhosis and bacterial infections in a tertiary center in Northern Germany
Source: BMC Gastroenterol. 2021 Jul 20;21:296. doi: 10.1186/s12876-021-01871-w (PMC8290615; doi:10.1186/s12876-021-01871-w)
Supplement: Supplementary file 2 — Additional file 2. Supplemental table 2: Antimicrobial categories and agents used to define MDR. [file 12876_2021_1871_MOESM2_ESM.docx]

**Supplemental table 2:** Antimicrobial categories and agents used to define MDR.

| **Antimicrobial category** | **Antimicrobial agent** | | | |
| --- | --- | --- | --- | --- |
|  | **Staph. aureus** | **Enterococcus spp.** | **Enterobacteriaceae** | **Acinetobacter spp.** |
| Aminoglycosides | Gentamicin | Gentamicin (high level) | Gentamicin  Tobramycin  Amikacin  Netilmicin^b^ | Gentamicin  Tobramycin  Amikacin  Netilmicin^b^ |
| Streptomycin |  | Streptomycin (high level) |  |  |
| Ansamycins | Rifampin/rifampicin |  |  |  |
| Anti-MRSA cephalosporins | Ceftaroline |  | Ceftaroline |  |
| Anti-staphylococcal β-lactams (or cephamycins) | Oxacillin (or cefoxitin) |  |  |  |
| Antipseudomonal carbapenems |  |  |  | Imipenem  Meropenem  Doripenem |
| Antipseudomonal fluoroquinolones |  |  |  | Ciprofloxacin  Levofloxacin |
| Antipseudomonal penicillins + β-lactamase inhibitors |  |  | Ticarcillin-clavulanic acid^b^  Piperacillin-tazobactam | Ticarcillin-clavulanic acid^b^  Piperacillin-tazobactam |
| Carbapenems^a^ |  | Imipenem  Meropenem  Doripenem | Ertapenem  Imipenem  Meropenem  Doripenem^b^ |  |
| Non-extended spectrum cephalosporins; 1^st^ and 2^nd^ generation cephalosporins |  |  | Cefazolin^a^  Cefuroxime |  |
| Extended-spectrum cephalosporins; 3^rd^ and 4^th^ generation cephalosporins |  |  | Cefotaxime or ceftriaxone  Ceftazidim  Cefepime | Cefotaxime  Ceftriaxone  Ceftazidim  Cefepime |
| Cephamycins^a^ |  |  | Cefoxitin  Cefotetan^b^ |  |
| Fluoroquinolones | Ciprofloxacin  Moxifloxacin | Ciprofloxacin  Levofloxacin  Moxifloxacin | Ciprofloxacin |  |
| Folate pathway inhibitors | Trimethoprim-sulphamethoxazole |  | Trimethoprim-sulphamethoxazole | Trimethoprim-sulphamethoxazole |
| Fucidanes | Fusidic acid |  |  |  |
| Glycopeptides | Vancomycin  Teicoplanin  Telavancin^b^ | Vancomycin  Teicoplanin |  |  |
| Glycylcyclines | Tigecycline | Tigecycline | Tigecycline |  |
| Lincosamides | Clindamycin |  |  |  |
| Lipopeptides | Daptomycin | Daptomycin |  |  |
| Macrolides | Erythromycin |  |  |  |
| Oxazolidinones | Linezolid | Linezolid |  |  |
| Monobactams |  |  | Aztreonam |  |
| Phenicols | Chloramphenicol |  | Chloramphenicol |  |
| Phosphonic acids | Fosfomycin |  | Fosfomycin |  |
| Penicillins^a^ |  | Ampicillin | Ampicillin |  |
| Penicillins + β -lactamase inhibitors^a^ |  |  | Amoxicillin-clavulanic acid  Ampicillin-sulbactam | Ampicillin-sulbactam |
| Polymyxins |  |  | Colistin | Colistin  Polymyxin B^b^ |
| Streptogramins^a^ | Quinupristin-dalfopristin | Quinupristin-dalfopristin |  |  |
| Tetracyclines | Tetracycline  Doxycycline  Minocycline^b^ | Doxycycline  Minocycline^b^ | Tetracycline  Doxycycline  Minocycline^b^ | Tetracycline  Doxycycline  Minocycline^b^ |

^a^ Antimicrobial category or agent was removed and not counted in species with intrinsic resistance against this antimicrobial category or agent ^b^ Not tested at our microbiology department, MDR: multidrug-resistance, Quoted from (28).
