# Supplementary material for: Antimicrobial resistance in patients with decompensated liver cirrhosis and bacterial infections in a tertiary center in Northern Germany
Source: BMC Gastroenterol. 2021 Jul 20;21:296. doi: 10.1186/s12876-021-01871-w (PMC8290615; doi:10.1186/s12876-021-01871-w)
Supplement: Supplementary file 3 — Additional file 3. Supplemental table 3: Pathogens in polymicrobial infections. [file 12876_2021_1871_MOESM3_ESM.docx]

**Supplemental table 3:** Pathogens in polymicrobial infections.

| **Culture site & patient** | | **Detected pathogens** |
| --- | --- | --- |
| Ascites:  4 pathogens | 1)  2) | Acinetobacter lwoffii, Enterococcus casseliflavus, S. epidermidis, S. haemolyticus  E. faecium, S. aureus, Acinetobacter lwoffii, S. haemolyticus |
| 3 pathogens | 1)  2)  3) | E. faecium, S. epidermidis, S. xylosus  S. aureus, S. haemolyticus, Corynebacterium amycolatum/xerosis  Acinetobacter lwoffii, S. auricularis, S. hominis |
| 2 pathogens | 1)  2)  3)  4)  5)  6)  7)  8)  9) | E. faecalis, K. pneumoniae  E. faecalis, S. epidermidis  E. faecalis, E. coli  E. coli, S. haemolyticus  K. pneumoniae, S. haemolyticus  E. faecium, S. haemolyticus  E. faecium, Candida kefyr  S. epidermidis, Streptococcus mitis-group  Bacillus sp., Paenibacillus sp. |
| Blood:  3 pathogens | 1)  2) | E. faecalis, K. pneumoniae, S. epidermidis  E. faecalis, E. faecium, E. coli. |
| 2 pathogens | 1)  2)  3)  4)  5)  6)  7)  8)  9)  10) | E. faecalis, E. coli  E. faecalis, S. aureus  E. faecium, S. epidermidis  S. aureus, Veillonella sp.  S. aureus, S. epidermidis  S. epidermidis, S. haemolyticus  S. epidermidis, Acinetobacter baumanii  K. pneumoniae, Stenotrophomonas maltophilia  Candida albicans, Candida glabrata  Candida albicans, Candida dubliensis |
| Urine:  4 pathogens | 1) | S. epidermidis, S. hominis, Lactococcus lactis, gram-positive coryneform bacillus |
| 3 pathogens | 1-2)  3)  4) | S. aureus, gram-positive and gram-negative mixed flora, yeasts  E. faecium, Candida glabrata, Candida albicans  K. pneumoniae, gram-positive and gram-negative mixed flora, Candida glabrata |
| 2 pathogens | 1-5)  6-10)  11)  12)  13)  14)  15)  16)  17)  18)  19)  20)  21)  22-23) | E. faecium, Candida albicans  E. faecalis, E. coli  E. faecalis, E. faecium  E. faecalis, K. pneumoniae  E. faecalis, K. oxytoca  E. faecalis, S. epidermidis  E. faecalis, viridans streptococci  E. coli, K. oxytoca  E. coli, K. pneumoniae  E. coli, Candida glabrata  S. aureus, Candida albicans  K. oxytoca, Candida tropicalis  gram-positive mixed flora, yeasts  Candida albicans, Candida glabrata |
