# Supplementary material for: Antimicrobial resistance in patients with decompensated liver cirrhosis and bacterial infections in a tertiary center in Northern Germany
Source: BMC Gastroenterol. 2021 Jul 20;21:296. doi: 10.1186/s12876-021-01871-w (PMC8290615; doi:10.1186/s12876-021-01871-w)
Supplement: Supplementary file 4 — Additional file 4. Supplemental table 4: Overview of the frequency and locations of infections. [file 12876_2021_1871_MOESM4_ESM.docx]

**Supplemental table 4:** Overview of the frequency and locations of infections.

|  | **Samples*** | **Positive samples***  (n, % of samples) | **Pathogens** | **MDR-bacteria**  (n, % of pathogens) | **Severe resistance**  (n, % of pathogens) |
| --- | --- | --- | --- | --- | --- |
| **Ascites** | 310 | 72 (23.2 %) | 90 | 22 (24.4 %) | 3 (3.3 %) |
| **Blood** | 98 | 55 (56.1 %) | 63 | 14 (22.2 %) | 3 (4.8 %) |
| **Urine** | 124 | 83 (66.9 %) | 108 | 24 (22.2 %) | 8 (7.4 %) |

*If a pathogen was detected repeatedly, only the first sample was counted. MDR: multidrug-resistance.
