# Supplementary material for: Antimicrobial resistance in patients with decompensated liver cirrhosis and bacterial infections in a tertiary center in Northern Germany
Source: BMC Gastroenterol. 2021 Jul 20;21:296. doi: 10.1186/s12876-021-01871-w (PMC8290615; doi:10.1186/s12876-021-01871-w)
Supplement: Supplementary file 5 — Additional file 5. Supplemental table 5: Susceptibility of pathogens in community-acquired vs. nosocomial infections. [file 12876_2021_1871_MOESM5_ESM.docx]

**Supplemental table 5:** Susceptibility in pathogens in community-acquired vs. nosocomial infections.

| **Antibiotic agent** | **Community-acquired SBP** | | | | **Nosocomial SBP** | | | | **p-value** |
| --- | --- | --- | --- | --- | --- | --- | --- | --- | --- |
|  | Susceptible | | Resistant | | Susceptible | | Resistant | |  |
|  | n | % | n | % | n | % | n | % |  |
| Ampicillin/sulbactam | 5 | 55.6 | 4 | 44.4 | 46 | 61.3 | 29 | 38.7 | 0.73 |
| Piperacillin/tazobactam | 6 | 66.7 | 3 | 33.3 | 49 | 65.3 | 26 | 34.7 | 1.00 |
| Cefuroxime | 3 | 37.5 | 5 | 62.5 | 34 | 47.2 | 38 | 52.8 | 0.72 |
| Ceftriaxone | 3 | 37.5 | 5 | 62.5 | 18 | 25.0 | 54 | 75.0 | 0.43 |
| Meropenem | 6 | 75.0 | 2 | 25.0 | 48 | 72.7 | 18 | 27.3 | 1.00 |
| Vancomycin | 9 | 90.0 | 1 | 10.0 | 54 | 72.0 | 21 | 28.0 | 0.44 |
| **Antibiotic agent** | **Community-acquired UTI** | | | | **Nosocomial UTI** | | | | **p-value** |
|  | Susceptible | | Resistant | | Susceptible | | Resistant | |  |
|  | n | % | n | % | n | % | n | % |  |
| Piperacillin/tazobactam | 5 | 83.3 | 1 | 16.7 | 31 | 63.3 | 18 | 36.7 | 0.65 |
| Amoxicillin-clavulanic acid | 2 | 66.7 | 1 | 33.3 | 10 | 43.5 | 13 | 56.5 | 0.58 |
| Cefpodoxime | 2 | 33.3 | 4 | 66.7 | 16 | 41.0 | 23 | 59.0 | 1.00 |
| Ceftriaxone | 2 | 25.0 | 6 | 75.0 | 20 | 40.8 | 29 | 59.2 | 0.47 |
| Fosfomycin | 0 |  | 0 |  | 15 | 88.2 | 2 | 11.8 |  |
| Levofloxacin | 3 | 60.0 | 2 | 40.0 | 29 | 59.2 | 20 | 40.8 | 1.00 |
| Ciprofloxacin | 2 | 100.0 | 0 | 0.0 | 23 | 71.9 | 9 | 28.1 | 1.00 |
| Meropenem | 2 | 66.7 | 1 | 33.3 | 29 | 85.3 | 5 | 14.7 | 0.42 |
| Cotrimoxazol | 2 | 33.3 | 4 | 66.7 | 25 | 50.0 | 25 | 50.0 | 0.67 |
| Nitrofurantoin | 0 |  | 0 |  | 10 | 100.0 | 0 | 0.0 |  |

SBP: spontaneous bacterial peritonitis; UTI: urinary tract infection; n: number of susceptible or resistant results.
